# Upregulation of bone morphogenetic protein 1 is associated with poor prognosis of late-stage gastric Cancer patients

**DOI:** 10.1186/s12885-018-4383-9

**Published:** 2018-05-02

**Authors:** Yung-Yu Hsieh, Shui-Yi Tung, Hung-Yu Pan, Chih-Wei Yen, Huang-Wei Xu, Yi-Fang Deng, Ying-Jhen Lin, Wan-Ting Hsu, Cheng-Shyong Wu, Chin Li

**Affiliations:** 1 Department of Gastroenterology and Hepatology, Chiayi Chang Gung Memorial Hospital, Chiayi, Taiwan, Republic of China; 2 College of Medicine, Chang Gung University, Taoyuan, Taiwan; 3 Department of Applied Mathematics, National Chiayi University, Chiayi, Taiwan; 4 Department of Life Science, National Chung Cheng University, 168 University Rd, Minhsiung, Chiayi, 62102 Taiwan, Republic of China

**Keywords:** Gastric cancer, Bone morphogenetic protein 1, Cell mobility

## Abstract

**Background:**

Gastric cancer is the eighth most common cancer in Taiwan, with a 40% 5-year survival rate. Approximately 40% of patients are refractory to chemotherapy. Currently, the anti-HER2 therapy is the only clinically employed targeted therapy. However, only 7% patients in Taiwan are HER2-positive. Identifying candidate target genes will facilitate the development of adjuvant targeted therapy to increase the efficacy of gastric cancer treatment.

**Methods:**

Clinical specimens were analyzed by targeted RNA sequencing to assess the expression levels of target genes. Statistical significance of differential expression and correlation between specimens was evaluated. The correlation with patient survival was analyzed as well. In vitro cell mobility was determined using wound-healing and transwell mobility assays.

**Results:**

Expression of BMP1, COL1A1, STAT3, SOX2, FOXA2, and GATA6 was progressively dysregulated through the stages of gastric oncogenesis. The expression profile of these six genes forms an ubiquitously biomarker signature that is sufficient to differentiate cancer from non-cancerous specimens. High expression status of BMP1 correlates with poor long-term survival of late-stage patients. In vitro, suppression of BMP1 inhibits the mobility of the gastric cancer cell lines, indicating a role of BMP1 in metastasis.

**Conclusions:**

BMP1 is upregulated in gastric cancer and is correlated with poor patient survival. Suppression of BMP1 reduced gastric cancer mobility in vitro. Our finding suggests that anti-BMP1 therapy will likely augment the efficacy of standard chemotherapy and improve the treatment outcome.

## Background

Gastric cancer is the eighth most common cancer in Taiwan, and approximately 90% of gastric cancer cases reported to the Taiwan Cancer Registry are adenocarcinomas [1]. Despite recent advances in treatment, the prognosis of late-stage gastric cancer is still very poor, and the 5-year relative survival rate is at approximately 40% with no significant improvement in the past decade. Annually, gastric cancer causes more than 2300 deaths [1]. Multiple risk factors are associated with higher risk of gastric cancer. Besides *Helicobacter pylori* (*Hp*) infection [2, 3], additional environmental and habitual factors, such as consumption of high-salt and fermented dietary products and smoking, are also associated with an increase of the risk for gastric cancer [4, 5]. Due to these diverse lifestyle-dependent risk factors, it is likely that the underlying oncogenic molecular mechanisms of gastric cancer display distinct biomarker signatures unique to different cultural populations.

Gastric adenocarcinomas was classified into intestinal, diffuse and mixed types by the histology-based Lauren system [6]. The 2010 WHO system provides a more refined classification system but is still based on histologic observation [7]. Advances in microarray and sequencing technology allowed development of classification systems based on the molecular biomarker signatures [8, 9]. Notably, Cancer Genome Atlas defined four types of gastric cancer based on infection of Epstein-Barr virus (EBV) and the status and type of genome stability [10]. However, there is no different treatment strategy that can be employed according to the molecular biomarkers. This disconnection between classification systems and treatment leads to little use of these molecular classification systems as treatment guidance.

Current gastric cancer treatment guideline is carried out regardless the result of histological or molecular typing. The primary treatment for gastric cancer is surgical resection, followed by adjuvant chemotherapy or radiation therapy [11]. For patients with limited invasion and undergoing partial or total gastrectomy, perioperative combinatory chemotherapy of fluorouracil, leucovorin, oxaliplatin, and docetaxel benefits the patients and extends average survival [12, 13]. But, for non-resectable, recurrent, or late-stage gastric cancer, the treatment outcome depends mainly on the efficacy of chemotherapy. Current combinatory chemotherapies, such as the combined use of 5-FU/leucovorin with either cisplatin or paclitaxel, can achieve a 50% to 60% response rate [14]. For patients with poor response to first-line chemotherapy or recurrent gastric cancer, second-line chemotherapy, including docetaxel and irinotecan, is only effective for a small percentage of patients [15, 16]. Although adjuvant targeted therapy enhanced the efficacy of conventional chemotherapy, the only approved targeted therapy for gastric cancer in Taiwan is anti-HER2 treatment [17, 18]. But less than 7% of patients in Taiwan are HER2-positive, and, as the result, only small number of gastric cancer patients benefits from targeted therapy. Given the limited use of targeted therapy in gastric cancer treatment, it is necessary to identify additional genes that can be therapeutically targeted in the majority of the patients.

The BMP signaling pathway plays essential role in development to ensure correct body patterning [19]. Bone morphogenetic protein 1 (BMP1), originally identified for its function in inducing cartilage formation in vivo, is not a ligand of the BMP signaling pathway but a secreted metalloprotease of the astacin metalloproteinase family [20]. The primary function of BMP1 is to cleaves the C-terminal of type I, II, and III procollagen to yield mature form for the formation of collagen fibrils [21, 22]. In addition to procollagens, BMP1 also cleaves laminins, hence involving in the basal lamina formation and maintenance [22]. Besides extracellular matrix protein processing, BMP1 also functions in the activation of the BMP signaling pathway. The availability of the BMP ligands are controlled by the BMP antagonists, including Noggin, Chordin, Follistatin, and Gremlin [23, 24]. BMP1 cleaves Chordin and releases BMP4 from the inhibitory interaction, thereby activating the BMP4 signaling pathway [23, 25]. Activation of transforming growth factor β (TGF-β) signaling pathway also requires BMP1 activity. TGF-β is produced as a part of a large precursor, pre-pro-TGF-β. It is processed during vesicular transport to produce TGF-β and latency-associated peptide (LAP). TGF-β and LAP interact to form a complex, in which LAP is then covalently linked to latent transforming growth factor beta binding protein 1 (LTBP1) to form the large latent complex (LLC). Once secreted, LLC is organized into the extracellular matrix, resulting in sequestering of TGF-β [26]. To release TGF-β from the matrix, BMP1 makes the initial cleavage of LTBP to release the LAP-TGF-β, which is in turned processed by other metalloproteinases such as MMP2 to free TGF-β [27]. Hence, BMP1 regulates the activation of the TGF-β and BMP signaling pathways by controlling the bioavailability of the ligands.

Here, we report the identification of a common biomarker signature in the gastric cancer patients from the southwest region of Taiwan. Among the dysregulated genes in this biomarker signature, upregulation of BMP1 was associated with poor survival of late-stage patients. Inhibition of BMP1 suppressed mobility in gastric cancer cell lines, suggesting that BMP1 upregulation may increase cancer invasiveness. Our findings could serve as the foundation for developing new prognostic markers and eventually leading to better treatment efficacy.

## Methods

### Targeted RNA sequencing and RNA sequencing

Biopsies collected during endoscopic examination were immersed overnight in RNAlater (Thermo Fisher Scientific, Waltham, MA) and stored at − 80 °C. Biopsies and frozen specimens were ground in TRI reagent (Thermo Fisher Scientific) and centrifuged to remove undissolved debris. Total RNA was extracted from biopsies and frozen specimens using TRI reagent (Thermo Fisher Scientific, Waltham, MA) according to the manufacturer’s protocol. The integrity and concentration of purified RNA samples were determined by capillary electrophoresis and fluorometric quantification. Sequencing-ready libraries of amplified targeted genes was prepared using TruSeq targeted RNA expression kits (Illumina, San Diego, CA, USA) with preassembled cell cycle and stem cells panels (Illumina). Sequencing was carried in a Miseq sequencer (Illumina), and the sequencing reads were remapped to the human genome (hg19) using a DEseq package. The expression level of each gene was represented by gene read number/total specimen read number. The libraries for RNA sequencing analysis were prepared using a SureSelect strand-specific mRNA library preparation kit (Agilent, Santa Clara, CA, USA). Libraries were sequenced in a Miseq sequencer. Mapping, annotation, and calculation of gene expression level (fragments per kilobase of transcript per million mapped reads, FPKM) was performed using CLC Genomic Workbench v. 8.5 (Qiagen, Redwood City, CA, USA). The sequencing data was deposited in Gene Expression Omnibus, National Center for Biotechnology Information, U.S.A. The accession numbers for targeted RNA sequencing and RNA sequencing are GSE80389 and GSE80388, respectively.

### Quantitative reverse transcription PCR (qRT-PCR)

Gastric cancer cell line AGS [28] (BCRC number 60102) was obtained from Bioresource Collection and Research Center, Hsinchu, Taiwan. MKN28 and MKN45 [29] was obtained from Dr. Michael Chan, Department of Biomedical Sciences, National Chung Cheng University, Taiwan. Use of these cell lines was approved by the Biosafety Committee of both Chiayi Chang Gung Memorial Hospital and National Chung Cheng University. The gastric cancer cell lines were cultured in RPMI 1640 medium supplemented with 10% fetal bovine serum (Sigma-Aldrich, St. Louis, MO, USA). Total RNA was purified from the cultured cells using TRI reagent (Thermo Fisher) according to the manufacturer’s protocol. cDNA was subsequently prepared from total RNA using MMLV high-performance reverse transcriptase (Illumina, San Diego, CA, USA) and oligo(dT) as the primer. Quantitative RT-PCR was performed using GoTag qPCR master mix (Promega, Madison, WI, USA) in a MiniOpticon PCR system (Biorad, Hercules, CA, USA). The primer sequences are 5’-CGACAGTCAGCCGCATCTTC and 5’-CCCAATACGACCAAATCCGTTGA for GAPDH, 5’-CAGTTTGACTTCTTTGAGACAGAGGGC and 5’-TGTGAGTCCACTGCGCACCTCCACG for BMP1, and 5’-GGTTTCCAATGTGTTCAATAGAT and 5’-CAATGCGGCTGTGAGTC for SERPINE1. The condition for 40 cycles of amplification was template denaturing at 94 °C for one minute, primer annealing at 55 °C for 30 s, and product extension at 72 °C for 45 s.

### Immunoblotting

The protein samples were prepared by directly dissolving the cells in 2% SDS loading buffer. The samples were separated by SDS-polyacrylamide gel electrophoresis and transferred to a Hybond-P membrane (GE Healthcare Life Sciences, Waukesha, WI, USA). The protein-bound membrane was blocked in 5% skim milk in phosphate-buffered saline (PBS) containing 0.1% Tween 20, followed by hybridization of the primary antibodies overnight at 4 °C. The primary antibodies were anti-BMP1 (Sigma-Aldrich, HPA014572) and anti-α-tubulin (Sigma-Aldrich, T5168) antibodies. The secondary antibodies used for detection were horseradish peroxidase-conjugated anti-mouse and anti-rabbit goat polyclonal antibodies (Sigma-Aldrich, A9044 and A0545). After hybridization of the secondary antibodies and extensive wash, chemiluminescence detection was performed using the Immobilon Western Chemiluminescent HRP Substrate (EMD Millipore, Billerica, MA, USA).

### Wound-healing and transwell assays

Gastric cancer cell lines AGS, MKN28, and MKN45 were seeded in the culture inserts. After attachment, cells were cultured in medium with 2% serum and small molecular inhibitor UK 383367 or Galunisertib (Selleck Chemicals, Houston, TX, USA) for 16 h. Inserts were removed and images were taken at 0 and 8 h using an Olympus FV1000 laser-scanning microscope. For each treatment and time point, three images were analyzed using ImageJ to obtain the mean length of the gap between migrating cells. The length at time 0 was defined as 100%, and the migration distance was the mean length that had been covered by migrating cells. The transwell assay was performed by seeding the cells into the upper chamber in the presence or absence of UK 383367 for 24 h. Cells were then cultured in the medium with no serum for an additional 24 h. Cells were fixed in 4% paraformaldehyde and stained with DAPI. Cells in the upper chamber were removed, and cells that had migrated into the lower chamber were imaged. Images were randomly taken using an Olympus FV1000 laser-scanning microscope before and after the cells in the upper chamber were removed. The images were analyzed using ImageJ to determine the cell number. The cell numbers from 5 images were added together. The migrated cells were calculated as the cells in the lower chamber divided by the total cells.

### Statistical analysis

Statistical analysis was carried out using an SAS Enterprise 5.1 statistical package (SAS Institute, Cary, NC, USA). Appropriate statistical methodology was selected according to distribution normality and sample variance to determine the significance of differential gene expression between two independent groups. When data in the compared groups displayed a normal distribution, a pooled *t*-test and Satterthwaite t-test were employed to calculate the significance of the data with and without equal variances, respectively. If the data were not normally distributed, a Wilcoxon rank-sum test is used to determine the significance of differential expression. When two groups are dependent, paired t-test and Wilcoxon signed rank test were employed with and without normal distribution, respectively. Multidimensional scaling (MDS) was performed for quality assessment and to explore the relationship between samples, based on Euclidean distances calculated from the regularized-logarithm transformation (rlog) transformed counts by the DEseq package. Survival was estimated using Kaplan-Meier analysis.

## Results

### Identifying a molecular signature of gastric cancer

In this study, we set out to screen for abnormally expressed genes in the clinical specimens in order to identify potential treatment targets. Our patient cohort consists of 6 normal, 14 chronic gastritis, 14 stage I/II cancer, and 24 stage III/IV cancer patients enrolled at the Chiayi Chang Gung Memorial Hospital. The patients’ basic information is summarized in Table 1. Since the integrity of RNA from clinical specimens was frequently compromised, we thus employed preassembled gene panels for targeted RNA sequencing to analyze the samples on the Illumina platform. This methodology enables high-throughput analysis of multiple genes on low-quality RNA samples. The reads from the sequencing runs were remapped to human genome hg19 using DEseq package. Expression level of each gene was normalized against total specimen read number and represented as gene read number/specimen read number.

**Table 1:** The age and sex of the study cohort. The study cohort consisted of 6 normal, 14 gastritis/IM, 14 early-stage (stage I/II) and 24 late-stage (stage III/IV) patients

	all	female	male	age
normal	6	4	2	39.8 ± 14.0
gastritis/IM	14	4	10	49.6 ± 20.4
carcinoma	38	11	27	69.8 ± 10.0
stage I	6	2	4	68.7 ± 5.8
stage II	8	2	6	72.9 ± 10.0
stage III	19	6	13	70.5 ± 9.6
stage IV	5	1	4	63.4 ± 15.0

**Fig. 1:**
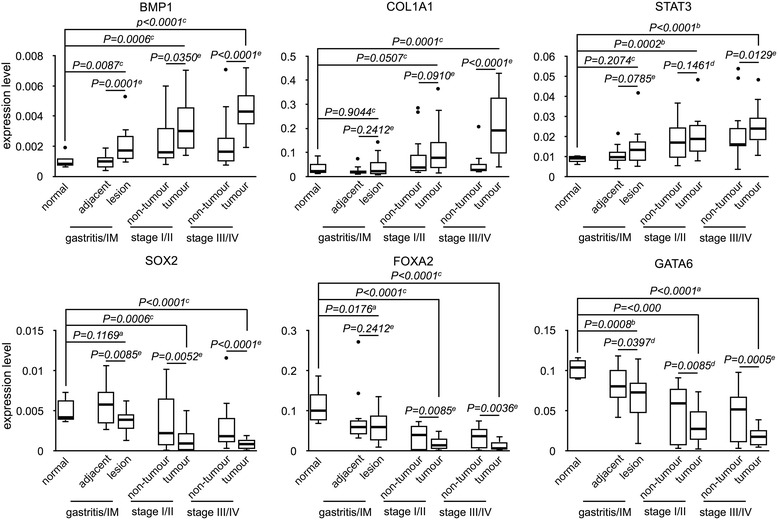
Expression of BMP1, COL1A1, STAT3, GATA6, FOXA2, and SOX2 is dysregulated in gastric cancer. The specimens of normal gastric epithelium as well as adjacent normal and lesion of gastritis and gastric tumor were analyzed by targeted RNA sequencing. Statistical significance analysis: a pooled t-test; b Saterthwaite t-test; c Wilcoxon rank-sum test; d paired t-test; e Wilcoxon signed rank test

**Fig. 2:**
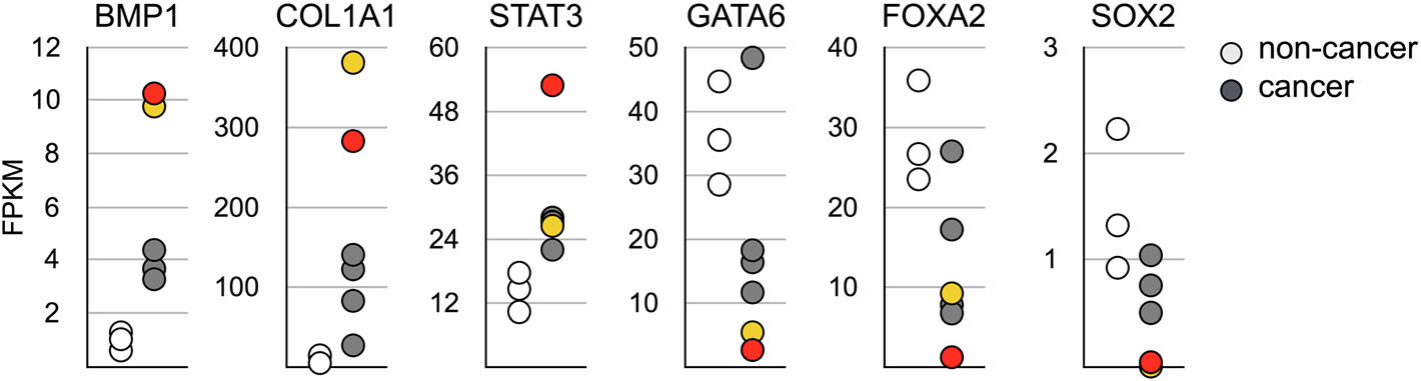
Dysregulation of BMP1, COL1A1, STAT3, GATA6, FOXA2, and SOX2 was confirmed by RNA sequencing. RNA expression analysis was performed on 3 normal gastric epithelium and 6 gastric tumor specimens. The gene expression level is represented using FPKM. Patients with highest and second highest BMP1 expression level are labeled in red and yellow, respectively

For statistical analysis, we divided the study cohort into early-stage (stage I and II) and late-stage (stage III and IV) groups. To avoid false identification due to insufficient sequencing depth, genes with marginal expression (expression < 0.001) were excluded from subsequent analysis. In addition genes dysregulated both in adjacent normal and cancer tissues were not further analyzed as well. Through rigorous screening, we identified six genes with expression change specifically in the lesions of cancer patients. Among these six genes, the expression level of bone morphogenetic protein 1 (BMP1), collagen 1A1 (COL1A1), and signal transducer and activator of transcription 3 (STAT3) is continuously increased from gastritis to the progression of cancer, while expression of GATA-binding factor 6 (GATA6), SRY-bo× 2 (SOX2) and forkhead box protein A2 (FOXA2) shows a progressively decreased trend (Fig. 1). To further confirm the result of targeted RNA sequencing, we performed transcriptome analysis on 3 non-cancer and 6 cancer samples. Consistent with the result of targeted RNA sequencing, RNA sequencing showed that BMP1, COL1A1, and STAT3 was upregulated and GATA6, SOX2, and FOXA2 was downregulated in the majority of the specimens (Fig. 2). Particularly, two patients showed significantly higher induction of BMP1 expression (Fig. 2, labeled in red and yellow circles). For these two patients, we also found the highest level of COL1A1 as well as the lowest level of GATA6 and SOX2, suggesting a strong correlation between the expression levels of these genes. Correlation analysis on the targeted RNA sequencing result also showed a similar phenomenon. A high level of BMP1 and STAT3 is simultaneously accompanied with an increase of COL1A1 and the suppression of GATA6, SOX2, and FOXA2.

**Fig. 3:**
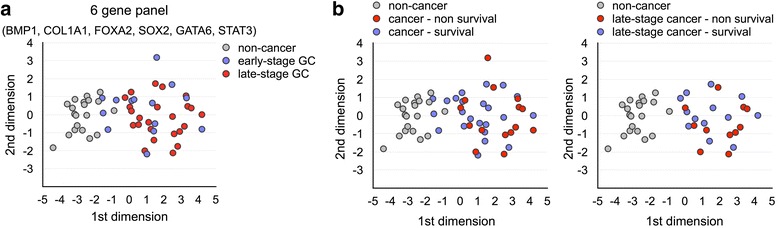
Dysregulation of BMP1, COL1A1, STAT3, GATA6, SOX2, and FOXA2 is a molecular signature of gastric cancer. (**a**) MDS analysis using the expression levels of BMP1, COL1A1, STAT3, GATA6, SOX2, and FOXA2 was performed to differentiate the cancer from non-cancer specimens. Non-cancer patients are represented in grey, and early-stage and late-stage patients were represented in blue and red, respectively. (**b**) MDS analysis was performed on all or late-stage patients. Survived cancer patients were represented in blue, and non-survived cancer patients were represented in red

We next investigate whether the expression profile of these six genes is sufficient to represent a molecular signature of gastric cancer. Multidimensional scaling analysis showed that the expression pattern of these six genes differentiated most of the gastric cancer from normal and gastritis specimens with few exceptions (Fig. 3a). The exceptions were few early-stage patients with a signature more close to that of non-cancer patients. Hence, the six-gene biomarker signature is not only a unique but also prevalent signature in our gastric cancer cohort. We then appended the information of patient survival to the multi-dimensional scaling (MDS) plot. For determining the living status of patients, only those patients with confirmed deceased date were considered non-survival, while all other patients were labeled as survival. Although there is no distinct non-survival subgroup within the cancer patients (Fig. 3b), it appeared that cancer patients with more distant expression signature from the non-cancer patients have poorer probability of survival. Our analysis, thus, showed that abnormal expression of BMP1, COL1A1, STAT3, GATA6, SOX2 and FOXA2 forms an ubiquitous molecular signature of gastric cancer patients in Southwestern Taiwan.

### Association of BMP1 upregulation with poor prognosis of gastric cancer

**Fig. 4:**
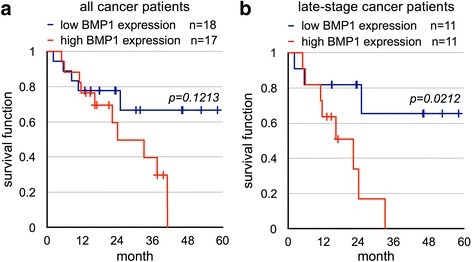
BMP1 upregulation is associated with poor prognosis for late-stage gastric cancer patients. The study cohort was grouped into high-BMP1- and low-BMP1-expressing groups, using the median level as the cut-off point. (**a**) Survival probability was calculated using Kaplan-Meier analysis on all cancer patients with at least a one-year follow-up. (**b**) Survival probability was calculated on late-stage patients only

Among the six genes in the molecular signature, small molecule inhibitors are currently available for BMP1 and STAT3 [30–32], making these two genes potential treatment targets. Activation of STAT3 induced by *H. pylori* infection was shown to promote gastric oncogenesis [33, 34], but the role of BMP1 in gastric cancer has not been examined before. Thus, we focused our investigation on the role of BMP1 in this study. The clinicopathological characteristics of patients and the BMP1 level at the adjacent and cancer lesion is listed in Table 2. However, no correlation between the level of BMP1 and the patients’ sex, *H. pylori* status, or cancer stage was identified. For survival analysis, cancer patients that just enrolled into the study was excluded, and the remaining patients were divided into two groups according to the status of BMP1 expression. The first group comprised patients with a BMP1 expression level above the median for all cancer specimens, while the other group had below-median levels of BMP1 expression. When all cancer patients were included in the analysis, the statistical significance of the survival probability between these two groups could not be established (Fig. 4a). Then, because early-stage patients generally have a much better treatment outcome and may obscure the effect of BMP1 upregulation, we re-analyzed the survival probability after excluding early-stage patients from the analysis. As expected, in the late-stage gastric cancer patients, the expression level of BMP1 correlated with survival outcome (Fig. 4b).

**Table 2:** Clinicopathological characteristics of the gastric cancer patients

age ranges	stage	TNM classificationTNM classificationTNM classification			Lauren’s classification	lympho-invasion	vaso-invasion	Tumor size	BMP1 at adjacent	BMP1 at lesion
75–79	IA	T1b	N0	Mx	intestinal	no	no	7 X 5 cm	0.00149	0.00708
70–74	IA	T1a	N0	Mx	mixed	no	no	1.2 X 0.5 cm	0.00099	0.00562
65–69	IA	T1b	N0	Mx	mixed	no	no	2.5 × 2 cm	0.00133	0.00305
60–64	IB	T2	N0	Mx	intestinal	yes	yes	4.5 × 4 cm	0.00291	0.00179
65–69	IB	T2	N0	Mx	mixed	no	no	6 X 4 cm	0.00075	0.00153
65–69	IB	T2	N0	Mx	mixed	no	no	2.2 × 2.1 cm	0.00288	0.00634
84–89	IIA	T3	N0	Mx	diffuse	no	no	5 × 4.5 cm	0.00411	0.00312
70–74	IIA	T3	N0	Mx	diffuse	no	no	1 × 1 cm	0.00114	0.00265
84–89	IIA	T3	N0	Mx	intestinal	no	no	7.5 X 7.0 cm	0.00147	0.00458
55–59	IIA	T2	N1	Mx	intestinal	no	no	7 X 6 cm	0.00122	0.00421
65–69	IIA	T3	N0	Mx	intestinal	no	no	4 X 3.5 cm	0.00117	0.00135
70–74	IIB	T2	N2	Mx	diffuse	yes	yes	4.5 × 2.0 cm	0.00251	0.00243
75–79	IIB	T2	N2	Mx	diffuse	yes	no	4.2 × 4.0 cm	0.00603	0.00457
60–64	IIB	T4a	N0	Mx	intestinal	no	no	2.6 X 2.0 cm	0.00167	0.00186
50–54	IIIA	T4	N1	Mx	diffuse	yes	no	8 X 6 cm	0.00466	0.00294
75–79	IIIA	T3	N2	Mx	mixed	yes	no	3.5 X 3.5 cm	0.00117	0.00404
60–64	IIIB	T3	N3	Mx	diffuse	yes	no	7 X 5 cm	0.00140	0.00471
65–69	IIIB	T4a	N2	Mx	diffuse	yes	yes	6 X 5 cm	0.00259	0.00552
75–79	IIIB	T3	N3b	Mx	intestinal	yes	no	3 X 2 cm	0.00353	0.00429
55–59	IIIB	T3	N3a	Mx	intestinal	yes	yes	3 X 3 cm	0.00708	0.00388
70–74	IIIB	T3	N3a	Mx	intestinal	yes	yes	body: 3.5 X 3.3 cm antrum: 3.8 X 3.5 cm	0.00098	0.00201
60–64	IIIB	T3	N3a	Mx	intestinal	yes	yes	3.5 X 3.0 cm	0.00181	0.00222
65–69	IIIB	T3	N3b	Mx	intestinal	yes	yes	5.5 × 4 cm	0.00199	0.00725
55–59	IIIB	T4b	N1	Mx	mixed	yes	no	8.5 X 7.5 cm	0.00082	0.00238
84–89	IIIB	T4a	N2	Mx	mixed	yes	no	3 X 2 cm	0.00314	0.00356
84–89	IIIB	T3	N3b	Mx	mixed	yes	yes	6.5 × 5 cm	0.00304	0.00443
70–74	IIIC	T4a	N3a	Mx	diffuse	yes	yes	7 X 2 cm	0.00150	0.00551
70–74	IIIC	T4a	N3b	Mx	diffuse	yes	yes	body: 1.2 × 1.0 cm antrum: 2.0 × 2.0 cm	0.00189	0.00635
75–79	IIIC	T4a	N3a	Mx	diffuse	yes	yes	4.5 x 2.5 cm	0.00091	0.00522
75–79	IIIC	T4a	N3a	Mx	diffuse	yes	yes	2.0 × 1.8 cm	0.00466	0.00507
70–74	IIIC	T4a	N3a	Mx	diffuse	yes	yes	4.8 × 4.5 cm	0.00204	0.00345
70–74	IIIC	T4	N3a	Mx	intestinal	yes	no	4.5 X 4 cm	0.00110	0.00428
65–69	IIIC	T4a	N3b	Mx	mixed	yes	no	8 X 7.5 cm	0.00239	0.00490
75–79	IV	T4b	N3b	M1	diffuse	no	yes	3 X 3 cm	0.00092	0.00387
75–79	IV	T1b	N1	M1	intestinal	no	no	2.2 X 2.0 cm	0.00072	0.00353
45–49	IV	T4b	N3a	M1	mixed	yes	yes	6 X 3 cm	0.00071	0.00196
45–49	IV	T4a	N3a	M1	mixed	no	no	4.5 X 4.5 cm	0.00178	0.00189
70–74	IV	T3	N3b	M1	intestinal	yes	yes	12 × 10.5 cm	0.00137	0.00543

### BMP1 inhibitor suppresses mobility of BMP1-expression gastric cancer cell lines

**Fig. 5:**
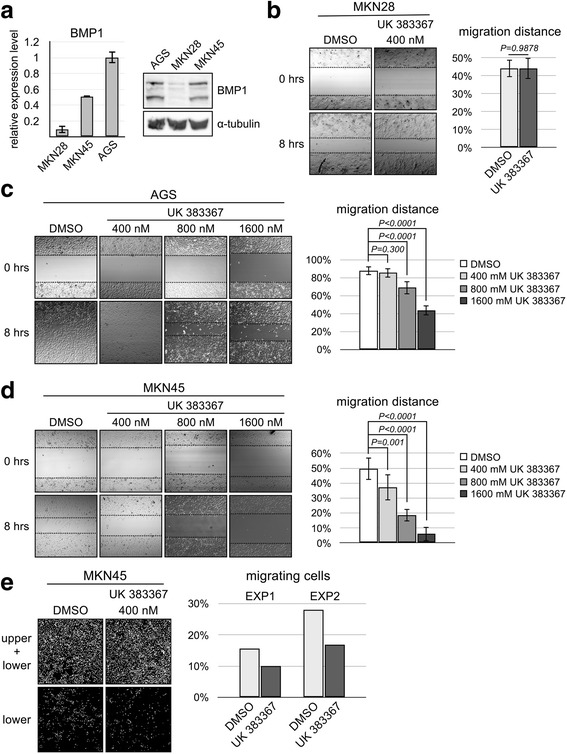
BMP1 inhibitor UK 383367 inhibits mobility of gastric cancer cell line AGS and MKN45. (**a**) Relative BMP1 expression of AGS, MKN28, and MKN45 cells was determined by qRT-PCR and immunoblotting. (**b**) MKN28 cells were seeded into a culture insert for 24 h, followed by culturing with 2% serum plus UK 383367 for another 24 h. After removal of the culture insert, images were taken 0 and 8 h using an Olympus FV1000 laser confocal scanning microscope equipped with differential interference contrast system. ImageJ was used to measure the distance covered by migrating cells, and statistical analysis was performed using Student’s t-test. (**c**) AGS cells was treated with 400 nM, 800 nM, or 1600 nM UK 3367, and the mobility of treated cells was assessed by wound healing assay as described in (**b**). (**d**) The mobility of MKN45 was assayed as described above. (**e**) MKN45 cells were seeded into the upper chamber of a transwell with or without UK 383367 and cultured for 24 h. Serum was then removed from the upper chamber, and the cells were allowed to move for 24 h. Cells were then fixed, stained with DAPI, and imaged using an Olympus FV1000 laser confocal scanning microscope. The percentage of the cells migrating to the lower chamber was calculated. Two independent experiments were carried out

Our data indicated that upregulation of BMP1 is correlation with poorer patient survival. In order to identify the role of BMP1 in the progression of gastric cancer, in vitro investigation was carried out using established gastric cancer cell lines. We first determined the expression level of BMP1 in gastric cancer cell lines AGS, MKN28, and MKN45 by quantitative RT-PCR and immunoblotting. The data showed that AGS and MKN45 expressed higher levels of BMP1 than MKN28 (Fig. 5a). Incidentally, these cell lines also displayed different morphology under the culture condition. While neighboring MKN28 cells adhere to each other and form cell islets, AGS and MKN45 were dispersed on the culture surface. To test the role of BMP1, the BMP1-specific suppressor UK 383367 was used in the assay to inhibit the BMP1 activity [30]. Initial examination showed that the growth rate of these cell lines was not significantly impacted by UK 383367, suggesting that upregulation of BMP1 does not promote cell growth (data not shown). We then performed a wound-healing assay to determine whether BMP1 inhibition led to decreased mobility. MKN28 had slowest mobility and lowest BMP1 expression, and its mobility was not significantly affected by the treatment with UK 383367 (Fig. 5b). In contrast, AGS and MKN45 had higher cell mobility and were progressively suppressed with increasing dosage of UK 383367 (Fig. 5c and d). In a transwell assay, UK 383367 also suppressed the movement of MKN45 cells from the upper to the lower chamber (Fig. 5e). Together, the result indicates that BMP1 inhibition reduces the mobility of gastric cancer cell line and supports the notion that BMP1 plays a role in metastasis of gastric cancer.

**Fig. 6:**
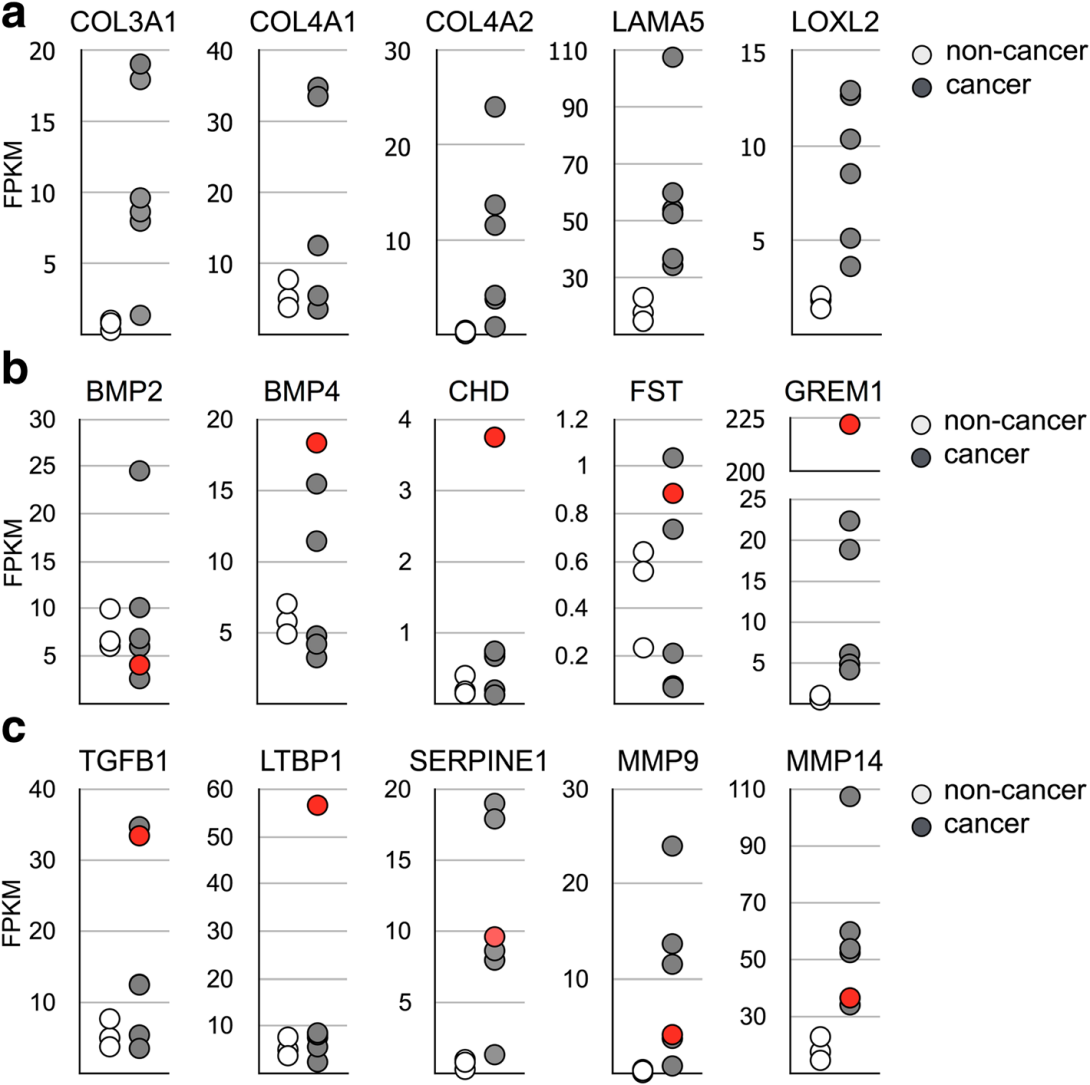
Extracellular matrix proteins were upregulated in gastric cancer. (**a**) The expression level of COL3A1, COL4A1, COL4A2, LAMA5, and LOXL2 in non-cancer and gastric cancer specimens was determined by RNA sequencing. (**b**) The expression level of BMP2, BMP4, Chordin, Follistatin, and Gremlin 1 in non-cancer and gastric cancer specimens was determined by RNA sequencing. (**c**) The expression level of TGF-β, LTBP1, SERPINE1, MMP9, and MMP14 in non-cancer and gastric cancer clinical specimens was determined by RNA sequencing

Functionally, BMP1 is the enzyme that initiates processing of procollagens and laminins into mature forms [21]. To determine whether BMP1 promotes cell mobility through modulating the extracellular matrix, we first analyzed the RNA sequencing data to determine the expression levels of extracellular matrix proteins. The result showed that there was a drastic dysregulation of many extracellular matrix proteins in gastric cancer. Among these genes, collagen 3A1 (COL3A1), collagen 4A1 (COL4A1), collagen 4A2 (COL4A2), and laminin 5 were upregulated in the majority of cancer lesions (Fig. 6a). COL3A1 is a type III collagen and a substrate of BMP1. COL4A1 and COL4A2 are type IV collagen, and they and laminin 5 are components of the basal lamina. In addition to increased expression of collagens and laminins, we also identified an increase of lysyl oxidase like 2 protein (LOXL2). Lysyl oxidase acts to crosslink matrix proteins and construct a firmer extracellular matrix, and its expression is negatively correlated with patient prognosis [35, 36]. In addition to extracellular matrix processing, BMP1 cleaves the bone morphogenetic protein 4 (BMP4) antagonist Chordin to increase the bioavailability of BMP4. RNA sequencing showed that two BMP ligands, bone morphogenetic protein 2 (BMP2) and BMP4, were expressed in gastric tissues. But, most cancer patients had similar expression level of BMP2 and BMP4 as non-cancer patients, indicating that the expression of BMP ligands was not altered in gastric cancer (Fig. 6b). The BMP antagonist Chordin and Follistatin was expressed only at marginal level in nearly all patients (Fig. 6b), and Noggin was not detected in all samples. The result suggests a limited role of Chordin, Follistatin, and Noggin in regulation of BMP signaling in gastric cancer. In contrast, Gremlin1 (GREM1) showed various degree of upregulation in cancer lesions (Fig. 6b). One patient displayed an increased expression of Chordin but also had highest level of GREM1 expression (Fig. 6b, labeled as red circle). Since GREM1 functions as an antagonist against both BMP2 and BMP4 [37], its upregulation would result in a general inhibition effect to the BMP signaling pathways in gastric cancer. Overall, our data indicates that the effect of BMP1 upregulation to cell functions is not mediated through activation of BMP signaling.

Besides the BMP signaling, BMP1 also cleaves TGF-β sequester LTBP1 to increase the level of free TGF-β ligand [27]. The result of targeted RNA sequencing indeed implied the possibility that the TGF-β pathway was highly activated in gastric cancer. This is because the expression of COL1A1 is activated by the TGF-β signaling [38, 39]. The BMP1 substrate LTBP1 was expressed at similar levels in all but only one cancer patient (Fig. 6c). In the patient with drastically increase of LTBP1, there was also an increase in TGF-β expression (Fig. 6c, labeled as red circle). Supporting evidence to the notion that TGF-β signaling is activated in gastric cancer was upregulation of additional TGF-β signaling target genes. Besides COL1A1, serpin family E member 1 (SERPINE1) [40], matrix metallopeptidase 9 (MMP-9) and matrix metallopeptidase 14 (MMP-14) [41, 42], were all drastically increased in cancer lesions as well (Fig. 6c). These genes were all reported to be upregulated by activated TGF-β signaling, adding to the evidences that the TGF-β signaling is activated in gastric cancer.

**Fig. 7:**
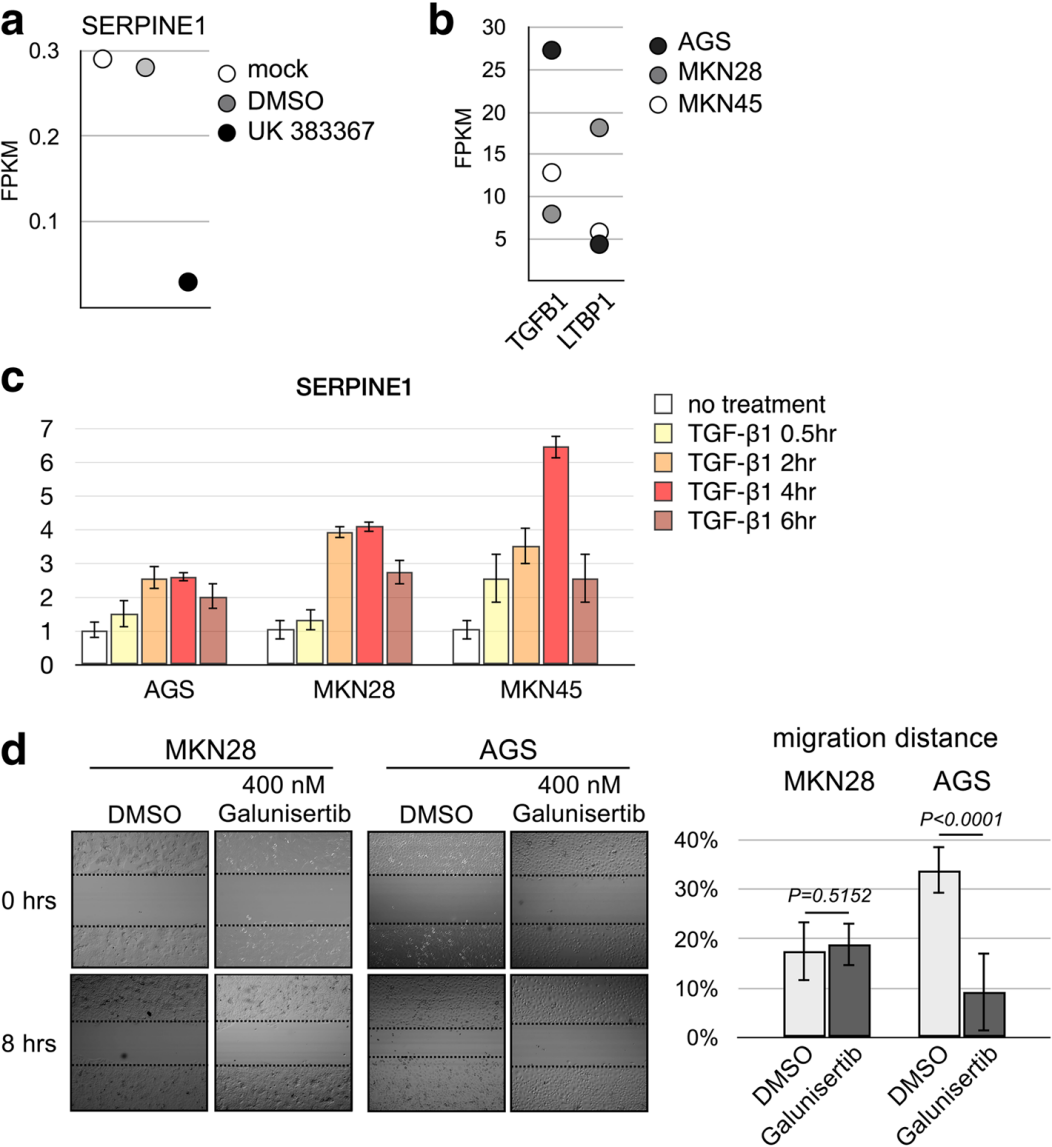
TGF-β inhibitor Galunisertib inhibits mobility of gastric cancer cell lines. (**a**) The level of SERPINE1 in mock-, DMSO-, and 400 mM UK 383367-treated MKN45 was determined by RNA sequencing. (**b**) The expression level of TGF-β and LTBP-1 in AGS, MKN28, and MKN45 was determined by RNA sequencing. (**c**) Total RNA was purified from AGS, MKN28, and MKN45 was treated with 10 ng/ml recombinant TGF-β for 0, 2, 4, and 6 h. The level of SERPINE1 was determine by qRT-PCR. (**d**) MKN28 and AGS were treated with 400 mM Galunisertib. The mobility was assessed using wound healing assay as described in Fig. 5b

The role of the TGF-β signaling pathway in cancer metastasis is well understood [43, 44]. We thus investigated whether the TGF-β signaling pathway plays a similar role in gastric cancer by in vitro experimentation. We first determined whether BMP1 regulated the activation status of the TGF-β signaling pathway in the established gastric cancer cell line. MKN45 was thus treated with UK 383367, and the expression profile was analyzed by RNA sequencing. The result showed that the treatment indeed reduced the expression level of SERPINE1 in MKN45 (Fig. 7a), suggesting that the suppression of cell mobility by the UK 383367 treatment was at least in part through suppression of the TGF-β signaling pathway. RNA sequencing also showed that AGS displayed highest TGF-β and lowest LTBP1 expression level (Fig. 7b). On the other hand, MKN28 showed lowest TGF-β but highest LTBP1 expression level (Fig. 7b).

We next evaluated whether the gastric cancer cell lines respond to an exogenous stimulus of TGF-β. The cells were treated with 10 ng/ml recombinant TGF-β for 2, 4, and 6 h, and the expression level of SERPINE1 was determined. The result showed that all three gastric cancer cell lines displayed an increase of SERPINE1 expression after TGF-β stimulation, and the increase of SERPINE1 peaked at 6 h post-treatment and started to decline (Fig. 7c). After confirming that the TGF-β was functional in these cells lines, we performed wound healing assays in the presence of Galunisertib [45, 46], a small molecule inhibitor of the TGF-β receptor I. Our data demonstrated that the mobility of AGS was suppressed by inhibition of the TGF-β pathway. However, although response to exogenous TGF-β stimulation, the mobility of MKN28 was not affected by the Galunisertib treatment (Fig. 7d). Our result indicated a potential use of either BMP1 or TGF-β inhibitors as a preventative or adjuvant treatment for TGF-β active gastric cancer.

## Discussion

Gastric cancer has a relatively poor 5-year survival rate. Although the efficacy of chemotherapy can be greatly improved by administration of adjuvant targeted therapy, only anti-HER2 therapy is currently being used for gastric cancer treatment in Taiwan. Due to low prevalence, only small number of patients benefit from the anti-HER2 therapy. In the current study, we identified ubiquitously dysregulated genes in the patient cohort from Southwestern Taiwan. The most prominent change is upregulation of BMP1, COL1A1, and STAT3 as well as downregulation of GATA6, SOX2, and FOXA2. Among these genes, deregulation of STAT3, GATA6, SOX2, and FOXA2 in gastric cancer was already reported previously in independent studies. Specifically, upregulation of STAT3 predicts poor outcome of gastric cancer [33, 34, 47]. Suppression of GATA6 in gastric cancer is associated with poor prognosis [48] and likely promotes tumorigenesis by reducing the expression of trefoil factor 1 (TFF1) and trefoil factor 2 (TFF2) [49]. Downregulation of SOX2 is a well-established and frequent event in gastric cancer and is mediated through epigenetic suppression [50, 51]. Expression of FOXA2, on the other hand, was shown to suppress gastric tumors [52], suggesting its role in gastric oncogenesis. Overall, our results are largely consistent with previous findings. Besides previously reported markers, first reported in this study is the drastic increase of BMP1 expression in gastric cancer. The expression level of BMP1 is statistically correlated with the survival of late-stage patients. The result of the wound healing assays indicates that the BMP1 upregulation contributes to an increase of cell mobility. These findings together suggests that overexpression of BMP1 contributes to cancer metastasis.

BMP1-dependent increase of cell mobility can be mediated through two possible mechanisms. One mechanism is an increase of extracellular matrix remodeling. Another possible mechanism is that increased cell mobility by BMP1 is through activation of signaling pathways. While our data shows that the BMP pathways were not activated, the TGF-β signaling appeared to be activated in gastric cancer. This notion was supported by activation of TGF-β target genes, including COL1A1, SERPINE1, MMP9 and MMP14 [38, 42]. The expression level of these genes may serve as an indicator of TGF-β signaling activation. The strong positive correlation between BMP1 and COL1A1 hence suggested the participation of BMP1 in TGF-β signaling activation. Most likely, upregulation of BMP1 will increase the bioavailability of TGF-β and subsequently activate the TGF-β signaling pathway. In summary, BMP1 upregulation could lead to rapid extracellular matrix remodeling and activate the TGF-β signaling. In addition, activated TGF-β signaling would enable immuno-evasion [53], contributing further to cancer growth and metastasis.

The effort to develop molecular classification systems demonstrates the heterogenous nature of gastric adenocarcinoma [7, 9, 10]. However, thus far, none of the molecular classification biomarkers and systems is widely adapted as regular part of clinical practice. This is because current treatment guideline does not call for distinct treatment toward different molecular types of gastric cancer. Hence, the molecular classification systems mainly provide only the information on the characterizations of gastric cancer but found limited use in patient treatment.

In this study, we identified a 6-gene molecular profile consisting the expression pattern of BMP1, COL1A1, STAT3, GATA6, SOX2, and FOXA2. Since our cohort is relatively small, additional study with larger cohort is certainly required to confirm this finding. In addition, our cohort consists of patients living in a rural agriculture area, this finding may be specific to the population with particular life style or dietary custom. Our statistical analysis was performed with the goal to find the most common expression alteration events. As the result, this molecular signature was found in the majority of patients and not correlated with clinicopathological characteristics, such as Lauren’s classification. Consequently, it is hence not useful as a classification criteria.

Although not applicable as a classification system, as a common feature, a therapy tailored against the genes in the signature can be adapted for more patients than current anti-HER2 therapy. Our data suggested the BMP1 likely promotes cancer cell migration through both faster extracellular matrix remodeling and activation of the TGF-β signaling. Currently, anti-BMP1 inhibitors are available, and STAT3 and TGF-β inhibitors are being tested for their efficacy against a variety of cancers in clinical trials. Used as combined therapy with current treatment regimen, these inhibitors may be able to reduce the risk of cancer metastasis and meaningfully extends patient survival.

## Conclusions

Our finding indicates that upregulation of BMP1 is correlated with the poor survival of gastric cancer patients. Our in vitro experiment demonstrated that UK 383367 reduces the mobility of gastric cancer cells. Conceivably, an anti-BMP1 therapy may be used as an adjuvant post-surgical therapy to reduce the risk of metastasis.

## Abbreviations

BMP1: Bone morphogenetic protein 1
BMP2: Bone morphogenetic protein 2
BMP4: Bone morphogenetic protein 4
COL1A1: Collagen 1A1
COL3A1: Collagen 3A1
COL4A1: Collagen 4A1
COL4A2: Collagen 4A2
EBV: Epstein-Barr virus
FOXA2: Forkhead box protein A2
FPKM: Fragments per kilobase of transcript per million mapped reads
GATA6: GATA-binding factor 6
GREM1: Gremlin1
*H. pylori*: *Helicobacter pylori*
LAP: TGF-β and latency-associated peptide
LLC: Large latent complex
LOXL2: Lysyl oxidase like 2 protein
LTBP1: Latent transforming growth factor beta binding protein 1
MDS: Multi-dimensional scaling
MMP14: Matrix metallopeptidase 14
MMP-9: Matrix metallopeptidase 9
SERPINE1: Serpin family E member 1
SOX2: SRY-bo× 2
STAT3: Signal transducer and activator of transcription 3
TFF1: Trefoil factor 1
TFF2: Trefoil factor 2
TGF-β: Transforming growth factor β
